# AC Electrokinetics of Polarizable Tri-Axial Ellipsoidal Nano-Antennas and Quantum Dot Manipulation

**DOI:** 10.3390/mi10020083

**Published:** 2019-01-24

**Authors:** Touvia Miloh

**Affiliations:** School of Mechanical Engineering, University of Tel-Aviv, Tel-Aviv 69978, Israel; miloh@eng.tau.ac.il

**Keywords:** AC electrokinetics, induced-charge electroosmosis, dielectrophoresis, ellipsoidal nano-antennas, fluorescence enhancement, quantum dot trapping

## Abstract

By realizing the advantages of using a tri-axial ellipsoidal nano-antenna (NA) surrounded by a solute for enhancing light emission of near-by dye molecules, we analyze the possibility of controlling and manipulating the location of quantum dots (similar to optical tweezers) placed near NA stagnation points, by means of prevalent AC electric forcing techniques. First, we consider the nonlinear electrokinetic problem of a freely suspended, uncharged, polarized ellipsoidal nanoparticle immersed in a symmetric unbounded electrolyte which is subjected to a uniform AC ambient electric field. Under the assumption of small Peclet and Reynolds numbers, thin Debye layer and ‘weak-field’, we solve the corresponding electrostatic and hydrodynamic problems. Explicit expressions for the induced velocity, pressure, and vorticity fields in the solute are then found in terms of the Lamé functions by solving the non-homogeneous Stokes equation forced by the Coulombic density term. The particular axisymmetric quadrupole-type flow for a conducting sphere is also found as a limiting case. It is finally demonstrated that stable or equilibrium (saddle-like) positions of a single molecule can indeed be achieved near stagnation points, depending on the directions of the electric forcing and the induced hydrodynamic (electroosmotic) and dielectrophoretic dynamical effects. The precise position of a fluorophore next to an ellipsoidal NA, can thus be simply controlled by adjusting the frequency of the ambient AC electric field.

## 1. Introduction

The subject of plasmonic luminescence enhancement and quenching of dye molecules or quantum dots (QD) placed near metallic nanoparticles (NP) or nano-antennas (NA), has recently attracted much interest because of its immense potential applications in the field of nano-photonics and nano-plasmonics (for example [1,2,3,4,5,6,7,8,9,10]). Light emitters in general are single atoms, organic or dye molecules as well as artificial molecules such as semiconductors, fluorophores, and quantum dots of a typical size of 10 nm or less, which is generally small compared to the characteristic length scale of the nearby polarizable NP (usually on the order of 100 nm). At these scales, it is prevalent to apply the electric or magnetic dipole (quasi-static) Rayleigh’s approximation to adequately describe the light-emission process of QD emitters placed next to a metallic nanostructure. While in this work, we will use the Rayleigh approximation, it should be stated that for a relatively large QD size, one needs to go beyond the common dipole approximation and consider for example higher-order multipole transitions [11] or a more intricate approach [12] such as Mie’s formulation [13].

**Figure 1:**
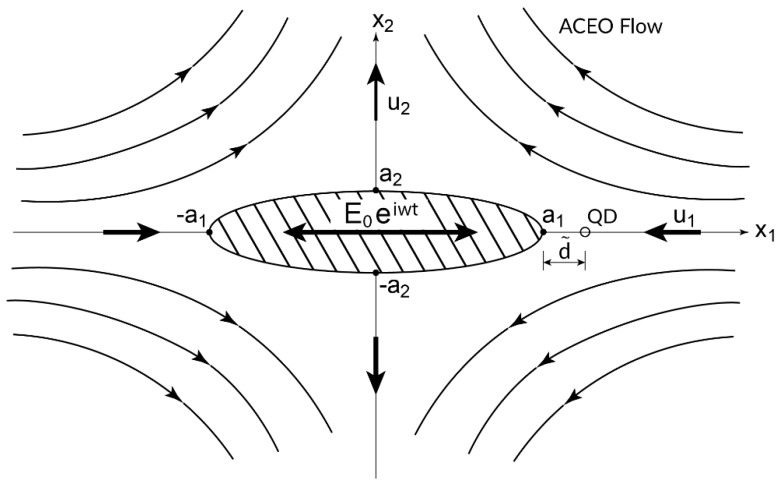
Schematic quadrupole ACEO flow field induced around a tri-axial ellipsoidal nano-antenna by an AC electric field acting along the longitudinal $x_{1}$ axis and displaying the position of a free QD at a distance $\tilde{d}$ from the nearest stagnation point.

The fluorescence rate (including enhancement and quenching) of a single-molecule lying next to an irradiated nano-antenna, depends on the morphology and plasmonic properties of the NP, the dielectric properties of the ambient electrolyte as well as on the size and photonic properties of the fluorophore (i.e., its absorption and emission bands) and mostly on the precise distance (position) of the QD near the NP [14,15,16,17,18,19]. The optimal QD/NP spacing for maximum fluorescence emission enhancement (Figure 1) is usually quite small (of the order of few nm) and exhibits a sharp maximum (peak) depending on geometry and dielectric parameters of both QD and NP. For example [14,15], for typical fluorophores or QDs located next to gold (Au) nano-spheres of diameters ranging from 20–100 nm that are illuminated at laser wave-length excitation of 400–650 nm (i.e., covering the blue, green, and red optical spectrum), the optimal QD/NP spacing is rather small and typically lies in the range of 8–15 nm. Maximum photonic enhancement at these optimal distances can be about five times larger compared to the ‘remote’ case. For larger spacing, the Gaussian fluorescence enhancement decays to unity, however if the distance between the QD and NA is relatively small (several nm), there is a strong quenching effect which implies that the non-radiative decay rate dominates the radiative part [1,3,5].

Among the major difficulties that one encounters when trying to implement such photoluminescence enhancement techniques is the ability to control and manipulate the precise location of a QD next to a plasmonic NP. One of the main contributions of the present work is a practical suggestion of using a simple electrokinetic-based method which combines common dielectrophoresis (DEP) and AC electroosmotic (ACEO) techniques [20,21,22,23,24] for the optimal positioning of free fluorophores next to NAs. The idea of actively controlling and manipulating the location of a single QD by optical means was probably first conceived by Ashkin’s [25] using the method of ‘optical tweezers’ and was later extended for the purpose of molecule and nanostructure trapping (see reviews by [26,27]). A similar procedure can be also employed for trapping QDs which are placed near planar electrodes by using DEP and ACEO effects, as demonstrated for example in [28,29,30] for the positioning of bio-functionalized semiconductor QDs (diameter less than 10 nm) on the center of the electrode or at the tip of aligned Au nanowires using AC electric fields with frequencies in the kHz range. Stable trapping was obtained when DEP and ACEO effects acting on a free QD are equal in magnitude and act in opposite directions. The optical-based approach works well for trapping micro-sized objects but possess some difficulties when trying to manipulate nanometric (atomic-sized) particles, because the amount of force exerted by light on such objects is rather small [29]. For this reason, electrokinetic-based methods using for example ambient AC electric fields seem to be more promising for QD positioning and trapping. Here, it is proposed to modify the same physical concept and implement it for the case of a single free QD placed near a polarizable (metallic) NP by applying a low-voltage uniform AC electric field. The size of the NP (or NA) is assumed to be large compared to that of the fluorophore (Figure 1) and the non-uniform electric field (unaffected by the presence of the QD) is induced around the NP due to its polarizability.

We employ the quasi-static (Rayleigh’s) approximation and represent the contribution of the free QD by a point dipole acting in the direction of the applied electric field. As a result of the field non-uniformity, the QD will experience a short-range DEP force which depends on the QD radius, its Clausius Mossotti (CM) coefficient, the forcing frequency of the imposed electric field and the local electric field gradient around the QD. In addition, the polarizability of the NP generates a tangential component of the electric field along the surface of the particle, which under the assumption of a thin Debye or electric double layer (EDL) results in a surface-slip velocity according to the Helmholtz Smoluchowski (HS) model [23] which drives a quadrupole-type ACEO motion in the solute (Figure 1). Since for typical micro-fluid applications, inertia effects can be neglected with respect to viscous ones, the long-range ACEO [24] or opto- [31,32] induced flow fields (forced by HS slippage), can be modeled by the Stokes momentum equation. Finally, treating the small-size QDs as free tracers, it is assumed that they are simply carried by the induced ACEO fluid motion (typical velocities of the order of few μm/s). The resulting ACEO force acting on the QD, is then determined by the fluid velocity (magnitude and direction) induced at the location of the QD and the resulting Stokes drag experienced by the QD.

It has been demonstrated that, in order to achieve simultaneous maximum enhancement and quenching emission rates, using tri-axial ellipsoidal NP shapes is superior to using common spherical morphologies, especially for practical photo-luminescence applications of fluorophores [5], since general orthotropic NAs have more than one resonance frequency and for this reason can in principle lead to a simultaneous photonic enhancement (or quenching) of both emission and absorption rates. This is in direct contrast to the more often studied case of metallic (conducting) nano-spheres, which have only a single plasmonic frequency determined by the corresponding Frohlich resonance condition (depending on its material) regardless of its size. In this context, most fluorophores are characterized by two distinct emission and absorption frequency bands which are separated by the Stokes drift [33] (e.g., typical wave lengths of 346 nm and 445 nm for Alexa 532-type fluorophore) and for this reason ellipsoidal NAs possessing at least two resonant frequencies, are considered more effective NAs compared to the commonly used spherical NAs. It is also worth mentioning that non-spherical laser-heated conducting nanoparticles immersed in electrolyte (unlike spherical NPs), are generally subjected to a non-vanishing self-induced thermoosmotic (quadrupole-type) flow driven by the Seebeck effect [32]. Thus, it is easier to manipulate and control optical-induced thermoosmotic flow fields about orthotropic (ellipsoidal) NPs compared to say around a perfectly symmetric spherical particle. Yet another practical reason for favoring ellipsoidal shapes is because they can generate stronger DEP gradients at relatively lower electric fields in comparison to spherical NPs (of same volume). Needless to say, that a spherical geometry can be considered as a special limiting case of the generalized tri-axial ellipsoid morphology that will be studied herein (as well as needles, disks, and nanowires).

It is apparent that determining the non-linear polarization induced flow field about a non-spherical polarizable NP, is definitely not a trivial task. Previously, an explicit solution has been presented only for a perfectly symmetric sphere by [34,35] in the case of a thin EDL and by [36] for finite EDL. This fact has inspired and motivated us to analyze the induced non-linear ACEO fluid motion problem around a general polarized ellipsoidal NP (under the common Rayleigh’s, ‘weak-field’, and thin EDL assumptions) and obtain an explicit new solution for the ACEO induced fluid velocity, pressure, and vorticity fields in the unbounded electrolyte surrounding a tri-axial ellipsoidal NP. The known quadrupole-type ACEO velocity field [34,35,36] for a perfectly symmetric spherical NP, is then obtained under a proper limit. It is also worth mentioning that the new (nonlinear) AC solution thus obtained for polarizable ellipsoidal morphologies screened by an EDL, is essentially different from the corresponding well-known linear electrostatic solution for a dielectric ellipsoid [37] (see also [21,38]) expressed in terms of the CM coefficients involving some elliptic integrals, obtained by ignoring electric double-layer screening effects (and thus no allowance for electroosmotic flow).

The structure of the paper contains therefore two parts: In Section 2, we present the theoretical background of the physical model, which includes the governing equations and boundary conditions. In Section 3 we analytically solve the electrostatic polarization problem of a general ellipsoidal NP under the common Poisson–Nernst–Planck (PNP) and ‘weak-field’ assumptions [23,34,36]. Then we provide, in Section 4, an explicit solution for the corresponding ACEO hydrodynamic problem, including a verification of the new tangled expressions for perfectly symmetric spherical and spheroidal morphologies (Section 5). In the second part of this paper (Section 6), we analyze the intrinsic problem of controlling and manipulating the location of a single free fluorophore (QD) placed next to a NA stagnation point, by employing the classical Rayleigh’s dipole approach. It is demonstrated that, depending on the direction of the applied AC electric field, the precise position of a free QD placed next to a NA, can be controlled and set at a certain stable or saddle-like trapping point in the solute, when the competing dynamical effects of DEP and ACEO act in different directions, or alternatively the QD may be trapped near one of the stagnation (stable) points of the NA when the above two effects augment each other. Towards this goal, we explicitly determine the DEP force exerted on a free QD in terms of the sign of its CM coefficient and forcing electric frequency, which serves as the single control parameter of the current fluorescence problem. It is finally demonstrated that, regardless of the sign of the CM coefficient, there exists a single frequency (generally below the Maxwell–Wagner frequency limit) that controls the precise spacing between the QD and the NA. Thus, the position (spacing) of a single free QD from any ellipsoidal NP, can be effectively controlled and manipulated by simply adjusting the frequency of the ambient electric field. This scenario is explicitly demonstrated for the case of a free biological/synthetic QD located near the forward stagnation point along the major axis of a tri-axial ellipsoid, where the ambient field is aligned in the same direction. Similar (simple) explicit expressions for such trapping points, can be also obtained for spherical and spheroidal morphologies. We conclude in Section 7 with a short summary and discussion of the analytic results and proposed methodology.

## 2. Theoretical Background

We consider a perfectly conducting (ideally polarized) metallic uncharged micro/nano tri-axial ellipsoid immersed in an unbounded symmetric binary electrolyte solution which is exposed to a uniform AC electric field acting in the direction of one of the ellipsoid three principal axes. Using a Cartesian coordinate system (*x*_1_,*x*_2_,*x*_3_) coinciding with the ellipsoid geometric centroid, its surface *S* can be simply expressed as $\sum_{j=1}^{3}{\left(x_{j}/a_{j}\right)}^{2}=1$, where $a_{1}\geq a_{2}\geq a_{3}$ denote the corresponding three semi-axes of the ellipsoid. The dimensionless amplitude (constant) of the ambient electric field (normalized with respect to the thermal scale given by the product of the Boltzmann constant $k_{B}$ and absolute temperature T) applied, say along the $x_{j}\left(j=1,2,3\right)$ direction, is $E_{0}$ and the forcing frequency is denoted by ω. The initially uncharged polarizable particle induces a spatial distribution of electric charges in the solute by virtue of the Poisson relation which depends on the amplitude of the AC ambient electric field and oscillates with time with the same frequency. Due to particle polarizability, the induced electric field in the electrolyte is essentially non-uniform and satisfies the equipotential boundary condition on S (assuming a perfectly conducting particle). The electric field engendered around the particle is proportional to the applied field and the resulting Coulombic force density induced in the solute is thus quadratic in $E_{0}$. The non-linear electric force density drives an ACEO (induced-charge electroosmotic) creeping flow around the particle [2,3] with velocity and pressure fields of a dispersive nature (i.e., depending on ω) which vary spatially like ${\left|E_{0}\right|}^{2}$ and decay to zero away from the NP. By assuming an incompressible Newtonian fluid and ignoring inertia with respect to viscous effects, one can solve the homogeneous Stokes equation and analytically determine the non-linear induced ACEO flow about a conducting tri-axial ellipsoid.

For the present non-dimensional analysis, it was found useful to follow the general formulation and scaling of parameters used for example in [39]. Thus, we consider a symmetric binary electrolyte and employ the so-called ‘standard model’ based on the linearized PNP equations for a ‘weak’ (compared to the thermal scale) ambient electric field. Consequently, we ignore effects associated with concentration polarization and surface conductance (i.e., assuming small Dukhin number) as well as neglecting ion convection (small Peclet number) with respect to electric migration and diffusion, which renders complete decoupling between the electrostatic and the hydrodynamic problems. Furthermore, for low forcing frequencies below the Maxwell–Wagner frequency limit [39], it is possible to express the dimensionless electric field as $\vec{E}=-\nabla \tilde{\phi }$, where ϕ˜(x1,x2,x3;t)=Re{ϕ(x1,x2,x3)eiωt} is the electric potential, t denotes time and Re represents the real part. In a similar manner, we express the volumetric induced charge density as Q˜(x1,x2,x3;t)=Re{Q(x1,x2,x3)eiωt}, where both ϕ (normalized by $\varphi_{T}=k_{B}T$) and Q (normalized with respect to $\varepsilon \varphi_{T}/a_{1}^{2}$ where ε denotes the solvent permittivity and $a_{1}$ is the reference length), are considered as dimensionless complex-value stationary functions.

Under these assumptions, the relations between the electric potential ϕ and induced-charge Q can be expressed [39] in terms of the dimensionless Debye scale $\lambda_{0}$ (normalized with respect to the major axis $a_{1}$) and ion diffusivity D (symmetric binary electrolyte) as

(1) $$\begin{array}{ccc} 2\phi =-{\left(\frac{\lambda }{\lambda_{0}}\right)}^{2}Q+\chi ; & {\left(\frac{\lambda_{0}^{}}{\lambda }\right)}^{2}=1+i\Omega ; & \Omega =\frac{\omega {\left(a_{1}\lambda_{0}^{}\right)}^{2}}{D} \end{array}$$

where χ represents the harmonic contribution $\left(\nabla^{2}\chi =0\right)$ to the electric potential ϕ in Equation (1) and Ω denotes the Maxwell–Wagner polarization dimensionless frequency. The operator ∇ in the sequel is made non-dimensional with reference to $a_{1}$. Equation (1) follows directly from the linearized versions of the PNP equations that can be casted into

(2) $$\lambda^{2}\nabla^{2}Q=Q=-2\lambda_{0}^{2}\nabla^{2}\phi$$

which implies that the charge density Q is governed by the Helmholtz equation. Supplementing Equation (2) we have two boundary conditions stating that the particle surface S is both equipotential (perfect conductor) and ion-impenetrable (zero ionic flux), i.e.,

(3) $$\begin{array}{ccc} \phi =const; & 2\frac{\partial \phi }{\partial n}+\frac{\partial Q}{\partial n}=0 & \mathrm{On}\;S \end{array}$$

where $\frac{\partial }{\partial n}$ denotes the normal derivative on S. Without loss of generality, one can choose the constant in Equation (3) to be zero and by virtue of, Equation (1) get

(4) $$\begin{array}{cc} \phi =0; & \frac{\partial \chi }{\partial n}=-i\Omega {\left(\frac{\lambda }{\lambda_{0}}\right)}^{2}\frac{\partial Q}{\partial n}. \end{array}$$

Closure of the electrostatic problem is finally obtained by assuming that far from the particle $\chi \to -2E_{0}(x_{n}/a_{1})$, where the non-dimensional ambient uniform electric field is taken to be parallel to the $x_{n}\left(n=1,2,3\right)$ direction.

Having obtained the solution for the electrostatic problem (i.e., ϕ, Q and χ), these parameters can then be used as the forcing (Coulombic) terms in the following non-homogeneous dimensionless Stokes equation [39]

(5) $$\begin{array}{ccc} \nabla^{2}\vec{u}=\nabla P+\frac{Q}{2\lambda_{0}^{2}}\nabla \phi ; & & \nabla \cdot \vec{u} \end{array}=0$$

The dimensionless solenoidal velocity field $\vec{u}\left(u_{1},u_{2},u_{3}\right)$ (normalized here with respect to $\varepsilon \varphi_{T}^{2}/\eta a_{1}$ where η is the solvent dynamic viscosity), denotes the polarization-induced velocity in the solute and P (normalized by $\varepsilon \varphi_{T}^{2}/a_{1}{}^{2}$), is the corresponding hydrodynamic pressure. Finally, by substituting Equations (1) and (2) in Equation (5), the latter can be expressed in terms of an effective pressure term $P^{*}$(defined below) as

(6) $$\begin{array}{ccc} \nabla^{2}\vec{u}=\nabla P^{*}+\frac{1}{4}{\left(\frac{\lambda }{\lambda_{0}}\right)}^{2}\nabla^{2}Q\cdot \nabla \chi ; & & P^{*}=P-\frac{1}{8}{\left(\frac{\lambda }{\lambda_{0}^{2}}\right)}^{2}Q^{2} \end{array}.$$

For general orthotropic shapes that possess three mutual planes of symmetry, the induced electric and electro-osmotic flow fields around a perfectly conducting particle are fore and aft symmetric and thus an initially uncharged particle remains stationary (no phoretic motion). Furthermore, by considering the salient EDL (electric double layer) screening effect, which is usually of the order of few tenths of a nanometer, to be small compared to the characteristic length scale $a_{1}$ of the particle (‘thin’ EDL assumption), the tangential induced electroosmotic velocity on the particle surface, in accordance with the classical HS slip formula and Murtsovkin’s [34] modification, can be readily obtained from Equation (6) by double integration as $\vec{u_{t}}\sim {\left(\lambda /2\lambda_{0}\right)}^{2}Q\nabla_{t}(\chi )$. Moreover, since the surface S is assumed to be equipotential Equations (1) and (4) finally renders

(7) $$\vec{u_{t}}≅\frac{1}{8}\nabla_{t}\left(\chi^{2}\right)\;\mathrm{On}\;S$$

where $\nabla_{t}=\nabla -\left(\vec{n}\cdot \nabla \right)$ represents the tangential gradient evaluated over the smooth polarizable surface S and $\vec{n}$ denotes the outward normal. In addition to the HS velocity slip boundary condition Equation (74) the impervious boundary condition, ${\vec{u}\cdot \vec{n}|}_{s}=0$ must be also enforced together with the requirement that $\vec{u}\to 0$ away from the NP.

## 3. The Electrostatic Problem of a Conducting Tri-Axial Ellipsoid

In this section, we present a new analytic solution for the nonlinear induced-charge electrostatic problem of an ideally polarizable ellipsoidal particle embedded in an unbounded solute due to AC ambient uniform electric field. Since we seek only time-independent streaming solutions (i.e., averaging over a period), the DC solution thus obtained does not depend on the native (uniform) surface charge of the particle. Once the electrostatic problem is solved, one can resolve the corresponding hydrodynamic (electroosmotic) flow problem (Section 4) and determine both the dielectrophoretic (DEP) and induced-charge electroosmotic flow (ACEO) which are exerted on a free QD located near one of the stagnation points of the ellipsoid. In order to solve for the ACEO flow field about a perfectly conducting ellipsoidal particle forced by the quadratic Coulombic density term under the assumption of a relatively ‘thin’ EDL Equation (6), one can following [34] consider the homogeneous (unforced) Stokes equation

(8) $$\begin{array}{ccc} \nabla^{2}\vec{u}=\nabla P; & & \nabla \cdot \vec{u}=0 \end{array}$$

which is subjected to

(9) $$\vec{u_{t}}=\begin{array}{ccc} \left|u_{t}\right|\vec{T}=\frac{1}{8}\left[\nabla -\left(\vec{n}\cdot \nabla \right)\right]\left(\chi^{2}\right); & & \vec{u}\cdot \vec{n} \end{array}=0\;\mathrm{On}\;S$$

where $\vec{T}$ denote a tangential vector to S. Supplementing the above mixed velocity boundary condition Equation (9), we also impose a proper decay of the velocity $\vec{u}$ at large distances from *S*.

In order to determine the ACEO velocity field $\vec{u}\left(x_{1},x_{2},x_{3}\right)$ and pressure distribution $P\left(x_{1},x_{2},x_{3}\right)$ around the polarized particle, we need first to analytically solve the electrostatic problem for both the electric potential $\chi \left(x_{1},x_{2},x_{3}\right)$ and the induced-charge density $Q\left(x_{1},x_{2},x_{3}\right)$. Note that χ is governed by the Laplace equation whereas Q satisfies the Helmholtz equation. It is recalled that a separable solution for the Laplace equation can be obtained by means of ellipsoidal harmonics and employing Lamé functions expressed in terms of the three orthogonal ellipsoidal coordinates. However, unlike for spherical shapes, such a procedure cannot be used in principle for ellipsoidal geometries when solving the corresponding Helmholtz equation. This difficulty can be bypassed for a thin EDL by assuming that (see [39,40]) $\frac{\partial Q}{\partial n}\sim -\frac{Q_{s}}{a_{1}\lambda }$, where $Q_{s}$ denotes the value of Q evaluated on S. Imposing next the equipotential boundary condition Equation (4) simply renders $Q_{s}={\left(\lambda /\lambda_{0}\right)}^{2}\chi$. Thus, when solving for the quadratic ACEO flow field about a metallic ellipsoidal particle under the assumption of a thin EDL, only the harmonic part of the polarization potential χ enters into the formulation.

Let us first solve the electrostatic problem for a uniformly applied AC electric field directed along one of the three semi-axes of the ellipsoid by employing a triply orthogonal ellipsoidal coordinate system (ρ,μ,ν), which is related to the dimensionless Cartesian one $\left(x_{1},x_{2},x_{3}\right)$ by (see for example [41,42]) the following transformations:

(10) $$\begin{array}{l} \begin{array}{cc} x_{1}^{2}=\frac{\rho^{2}\mu^{2}\nu^{2}}{h_{2}^{2}h_{3}^{2}}; & x_{2}^{2}=\frac{\left(\rho^{2}-h_{3}^{2}\right)\left(\mu^{2}-h_{3}^{2}\right)\left(h_{3}^{2}-\nu^{2}\right)}{h_{3}^{2}h_{1}^{2}} \end{array}\\ x_{3}^{2}=\frac{\left(\rho^{2}-h_{2}^{2}\right)\left(h_{2}^{2}-\mu^{2}\right)\left(h_{2}^{2}-\nu^{2}\right)}{h_{2}^{2}h_{1}^{2}} \end{array}$$

where

(11) $$\begin{array}{cc} \begin{array}{cc} h_{1}^{2}=a_{2}^{2}-a_{3}^{2}; & h_{2}^{2}= \end{array}a_{1}^{2}-a_{3}^{2}; & h_{3}^{2}=a_{1}^{2}-a_{2}^{2} \end{array}$$

such that $\begin{array}{cccc} h_{2}\geq h_{3}; & \infty >\rho^{2}\geq a_{1}^{2}; & h_{2}^{2}>\mu^{2}\geq h_{3}^{2}; & h_{3}^{2}\geq \nu^{2}\geq 0 \end{array}$.

Any separable (normal) solution of the Laplace equation expressed in terms of ellipsoidal coordinates Equation (10) which is regular at the origin (‘interior’) can be written as [41,42]

(12) $$E_{n}^{m}\left(\rho ,\mu ,\nu \right)=E_{n}^{m}\left(\rho \right)E_{n}^{m}\left(\mu \right)E_{n}^{m}\left(\nu \right)$$

where $E_{n}^{m}(x)$ represents the Lamé function of the first kind of order n and degree m≤2n+1. In a similar manner, a separable solution that decays at infinity (‘exterior’) can be expressed as

(13) $$F_{n}^{m}\left(\rho ,\mu ,\nu \right)=F_{n}^{m}\left(\rho \right)E_{n}^{m}\left(\mu \right)E_{n}^{m}\left(\nu \right)$$

where the Lamé function of the second kind is defined below as

(14) $$\begin{array}{ccc} F_{n}^{m}\left(\rho \right)=I_{n}^{m}\left(\rho \right)E_{n}^{m}\left(\rho \right); & & I_{n}^{m}\left(\rho \right)=\left(2n+1\right)\int_{\rho }^{\infty }\frac{ds}{{\left[E_{n}^{m}\left(s\right)\right]}^{2}\sqrt{\left(s^{2}-h_{2}^{2}\right)\left(s^{2}-h_{3}^{2}\right)}}. \end{array}$$

Consider for example the case of a uniform ambient electric field of amplitude $E_{0}$ applied along the $x_{j}$ direction (j=1,2,3). A general expression for $\chi_{j}\left(x_{1},x_{2},x_{3}\right)$ can then be postulated according to Equation (1) as;

(15) $$\begin{array}{ccc} \chi_{j}\left(x_{1},x_{2},x_{3}\right)=-\frac{2E_{0}}{a_{j}G_{j}}\left[E_{1}^{j}\left(\rho \right)-C_{j}F_{1}^{j}\left(\rho \right)\right]E_{1}^{j}\left(\mu \right)E_{1}^{j}\left(\nu \right) & & j=1,2,3 \end{array}$$

where $\begin{array}{ccc} G_{1}=h_{2}h_{3}; & G_{2}=h_{3}h_{1}; & G_{3}=h_{1}h_{2} \end{array}$. Clearly, at infinity (ρ→∞), $\chi_{j}\to -2E_{0}(x_{j}/a_{j})$ and thus the coefficient $C_{j}$ in Equation (15) can be determined explicitly by Equation (4) for a ‘thin’ EDL (or at low-frequency where $\lambda \to \lambda_{0}$) and using ${\frac{\partial Q}{\partial n}|}_{S}=-\frac{Q_{S}}{a_{1}\lambda }$, from the following Robin (mixed) boundary condition:

(16) $$\frac{\partial \chi }{\partial n}=\frac{1}{h_{\rho }}\frac{\partial \chi }{\partial \rho }=i\frac{\Omega }{\lambda }\chi .\;\mathrm{on}\;S$$

Here, $h_{\rho }\left(\rho ,\mu ,\nu \right)$ denotes the normal metric coefficient evaluated on the ellipsoidal surface [43]. $\rho =a_{1}$. Equation (16) can be further simplified yielding

(17) $$\begin{array}{ccc} {\frac{\partial \chi }{\partial \rho }|}_{S}=\frac{i\Omega^{*}}{a_{1}}\chi ; & & \Omega^{*}=\frac{r_{1}\Omega }{\lambda_{}}=\frac{\omega ra^{2}{}_{1}\lambda_{0}}{D} \end{array}\sqrt{1+\frac{i\omega a_{1}^{2}\lambda_{0}^{2}}{D}}$$

where $\Omega^{*}$ represents the non-dimensional RC forcing frequency and r is a dimensionless ‘geometric’ parameter [40] defined as the average value of $1/h_{\rho }$ taken over the ellipsoidal surface S, namely

(18) $$\frac{1}{r}=\frac{1}{S}\int_{S}\frac{ds}{h_{\rho }}=\frac{3\forall }{a_{1}S}$$

with $\forall =(4\pi /3)a_{1}a_{2}a_{3}$ denoting the volume of the ellipsoid (see Section 12.6 in [42]). Note that r=1 for a sphere and $r\sim a_{1}/a_{3}>1$ for a slender prolate spheroid.

Substituting next Equations (14) and (17) in Equation (15) renders

(19) $$C_{j}=\frac{i\Omega^{*}E_{1}^{j}\left(a_{1}\right)-a_{1}{\dot{E}}_{1}^{j}\left(a_{1}\right)}{i\Omega^{*}F_{1}^{j}\left(a_{1}\right)-a_{1}{\dot{F}}_{1}^{j}\left(a_{1}\right)}=\frac{i\Omega^{*}{\left(\frac{a_{j}^{}}{a_{1}}\right)}^{2}-1}{I_{1}^{j}\left(a_{1}\right)\left[i\Omega^{*}{\left(\frac{a_{j}^{}}{a_{1}}\right)}^{2}-1\right]+4\pi /\forall }$$

where the upper dot represents differentiation with respect to the argument (i.e., ${\dot{E}}_{1}^{j}\left(a_{1}\right)=\frac{\partial E_{1}^{j}(\rho )}{\partial \rho^{}}$ evaluated at $\rho =a_{1})$. Note also that the coefficient $C_{j}/\forall$ is dimensionless. Finally, in order to enforce the HS velocity slip condition Equation (7), we need to determine the value of ${\left(\chi_{j}\right)}^{2}$ on $\rho =a_{1}$. This quadratic can be readily obtained from Equation (15) as (no sum over j)

(20) |χj2E0|2=K(j)(xj/aj)2;K(j)=1−2Re{Cj}I1j(a1)+|CjI1j(a1)|2=[(1−∀I1j(a1)4π)2+(∀I1j(a1)4πΩ∗(aja1)2)2]−1

which provides the corresponding coupling term between the electrostatic and hydrodynamic problems in terms of a geometric parameter $K^{\left(j\right)}$ and the direction (j=1,2,3) of the applied electric field.

## 4. Hydrodynamic Problem

Our next task is to analytically solve the homogeneous Stokes equation (8) for the ACEO velocity field, which is subjected to the velocity-slippage Equation (9). For this purpose, let us assume the following expression for the velocity field ${\vec{u}}^{\left(n\right)}\left(u_{1}^{\left(n\right)},u_{2}^{\left(n\right)},u_{3}^{\left(n\right)}\right)$ that is induced by polarization due to uniform ambient electric excitation $E_{0}$ acting along the $x_{n}$ direction [44] (n=1,2,3)

(21) $$u_{i}^{\left(n\right)}\left(x_{1},x_{2},x_{3}\right)=\sum_{k=1}^{3}A_{k}^{\left(n\right)}\left(x_{k}\frac{\partial \psi_{k}}{\partial x_{i}}-\delta_{ik}\psi_{k}\right)+a_{1}B^{\left(n\right)}\frac{\partial \Phi^{\left(n\right)}{}^{}}{\partial x_{i}}$$

such that both $\psi_{k}\left(x_{1},x_{2},x_{3}\right)$ and $\Phi^{\left(n\right)}(x_{1},x_{2},x_{3})$ are harmonic functions (namely $\begin{array}{cc} \nabla^{2}\psi_{k}=0; & \nabla^{2}\Phi^{\left(n\right)}=0 \end{array}$) which decay away from S and $\delta_{ij}$ denotes the Kronecker delta. Here, $A_{k}^{\left(n\right)}$ and $B^{\left(n\right)}$ are dimensionless geometric coefficients to be determined depending on the direction (n=1,2,3) of the applied AC electric field. In particular, we choose for k=1,2,3

(22) $$\psi_{k}\left(\rho ,\mu ,\nu \right)=\frac{a_{1}^{2}F_{1}^{k}\left(\rho ,\mu ,\nu \right)}{G_{k}}=a_{1}^{2}x_{k}I_{1}^{k}\left(\rho \right)\;(\mathrm{no}\;\mathrm{sum}\;\mathrm{over}\;k)$$

since according to Equations (12) and (14) $E_{1}^{k}\left(\rho ,\mu ,\nu \right)=G_{k}x_{k}$ and $F_{1}^{k}\left(\rho ,\mu ,\nu \right)=I_{1}^{k}\left(\rho \right)E_{1}^{k}\left(\rho ,\mu ,\nu \right)$.

The slip velocity on the surface of the ellipsoid is proportional by virtue of Equation (7) and Equation (20) to the tangential gradient of the coordinate square $x_{k}^{2}$. For this reason, we make use of the following relation (see Equation (G.83) in [42]) which expresses the monomial of the second-degree evaluated on S$\left(\rho =a_{1}\right)$ in terms of ellipsoidal surface harmonics as

(23) $$\begin{array}{cc} 3{\left(\frac{x_{n}}{a_{n}}\right)}^{2}=1-\frac{E_{2}^{1}\left(a_{1},\mu ,\nu \right)}{E_{2}^{1}\left(a_{1}\right)\left(\wedge -\wedge^{\prime }\right)\left(\wedge -a_{n}^{2}\right)}+\frac{E_{2}^{2}\left(a_{1},\mu ,\nu \right)}{E_{2}^{2}\left(a_{1}\right)\left(\wedge -\wedge^{\prime }\right)\left(\wedge^{\prime }-a_{n}^{2}\right)}; & n=1,2,3 \end{array}$$

Here ∧ and $\wedge^{\prime }$ represent the two roots of the binomial equation $\sum_{k}^{3}{\left(\wedge -a_{k}^{2}\right)}^{-1}=0$, which are explicitly given in terms of the geometric parameters $\left(h_{1},h_{2},h_{3}\right)$ defined in Equation (11) as [42]

(24) $$\begin{array}{cc} \wedge -\wedge^{\prime }=\frac{2}{3}\sqrt{h_{1}^{4}+{\left(h_{2}h_{3}\right)}^{2}}; & \wedge +\wedge^{\prime }=2\left[a_{1}^{2}-\frac{1}{3}\left(h_{2}^{2}+h_{3}^{2}\right)\right] \end{array}.$$

The particular quadratic nature of the HS velocity slip condition combined with Equation (20) suggests then that the harmonic function $\Phi^{\left(n\right)}$ in Equation (21), can be chosen (up to a constant) as

(25) $$\begin{array}{l} \Phi^{\left(n\right)}\left(\rho ,\mu ,\nu \right)=\left[\frac{F_{0}^{1}\left(\rho ,\mu ,\nu \right)}{F_{0}^{1}\left(a_{1}\right)}-\frac{F_{2}^{1}\left(\rho ,\mu ,\nu \right)}{F_{2}^{1}\left(a_{1}\right)\left(\wedge -\wedge^{\prime }\right)\left(\wedge -a_{n}^{2}\right)}+\frac{F_{2}^{2}\left(\rho ,\mu ,\nu \right)}{F_{2}^{2}\left(a_{1}\right)\left(\wedge -\wedge^{\prime }\right)\left(\wedge^{\prime }-a_{n}^{2}\right)}\right] \end{array}$$

One can readily verify that the parenthesis in Equation (25) is non-dimensional and that it satisfies Equation (23) for $\rho =a_{1}$. It can also be shown by using Equations (22) and (25) that the velocity field Equation (21) vanishes at large distances (ρ→∞) from the polarizable particle. Furthermore, it is evident that the velocity vector Equation (21) is solenoid and automatically satisfies the continuity equation $\partial u_{i}^{\left(n\right)}/\partial x_{i}=0$, since both $\psi_{k}$ and $\Phi^{\left(n\right)}$ are harmonic functions. In addition, it is also possible to analytically determine the hydrodynamic pressure distribution $P^{\left(n)\right)}$ in the solute by substituting Equation (21) in the homogeneous Stokes momentum equation Equation (8) which simply leads to

(26) $$P^{\left(n\right)}\left(x_{1},x_{2},x_{3}\right)=2a_{1}^{}\sum_{k=1}^{3}A_{k}^{\left(n\right)}\frac{\partial \psi_{k}}{\partial x_{k}}+P_{0},$$

where $P_{0}$ denotes the ambient (constant) pressure.

What remains now is to explicitly determine the velocity field by obtaining the coefficients $A_{k}^{\left(n\right)}$ in (21) and $B^{\left(n\right)}$ in Equation (25) using the boundary conditions Equation (9). Let us first note that by combining Equations (22) with (21), the ACEO velocity field can be expressed in a vector form as [44]

(27) $${\vec{u}}^{\left(n\right)}(x_{1},x_{2},x_{3})=a_{1}^{2}\sum_{k=1}^{3}A_{k}^{\left(n\right)}x_{k}^{2}\nabla I_{1}^{k}\left(\rho \right)+a_{1}B^{\left(n\right)}\nabla \Phi^{\left(n\right)}$$

which implies that the first term on the right hand side of Equation (27) does not have any tangential component along S, since by definition $\nabla_{t}I_{1}^{k}(\rho )=0$ on $\rho =a_{1}$. Thus, by virtue of Equation (23) and Equation (25), one gets $\Phi^{\left(n\right)}\left(a_{1},\mu ,\nu \right)=3{\left(x_{n}/a_{n}\right)}^{2}+const$ and when combined together with Equations (7), (9), and (20), the coefficient $B^{\left(n\right)}$ can be finally resolved in terms of the parameter $K^{\left(n\right)}$ Equation (20), as

(28) $$B^{\left(n\right)}=\frac{E_{0}^{2}}{6}K^{\left(n\right)}.$$

Next, in order to enforce the impervious velocity boundary condition Equation (9) on the ellipsoid, we recall that the normal derivative on S can be expressed as $\frac{\partial }{\partial n}=\frac{1}{h_{\rho }}{\frac{\partial }{\partial \rho }|}_{\rho =a_{1}}$ and thus by multiplying the right hand side of Equation (27) by the normal vector $\vec{n}$ to S, renders

(29) $$a_{1}^{}\sum_{k=1}^{3}A_{k}^{\left(n\right)}x_{k}^{2}{\dot{I}}_{1}^{k}\left(a_{1}\right)+B^{\left(n\right)}\frac{\partial \Phi^{\left(n\right)}}{\partial \rho }=0\;\mathrm{On}\;\rho =a_{1}.$$

The second term on the left-hand side of Equation (29) can be accordingly expressed for any point $\left(a_{1},\mu ,\nu \right)$ on S following Equation (25), as

(30) $${\frac{\partial \Phi^{\left(n\right)}}{\partial \rho }|}_{\rho =a_{1}}=\left[\frac{{\dot{F}}_{0}^{1}\left(a_{1}\right)}{F_{0}^{1}\left(a_{1}\right)}-\frac{{\dot{F}}_{2}^{1}\left(a_{1}\right)E_{2}^{1}\left(a_{1},\mu ,\nu \right)}{E_{2}^{1}\left(a_{1}\right)F_{2}^{1}\left(a_{1}\right)\left(\wedge -\wedge^{\prime }\right)\left(\wedge -a_{n}^{2}\right)}+\frac{{\dot{F}}_{2}^{2}\left(a_{1}\right)E_{2}^{2}\left(a_{1},\mu ,\nu \right)}{E_{2}^{2}\left(a_{1}\right)F_{2}^{2}\left(a_{1}\right)\left(\wedge -\wedge^{\prime }\right)\left(\wedge^{\prime }-a_{n}^{2}\right)}\right]$$

In order to express Equation (30) in terms of monomials of the second-degree, we make use of the following identities (see Equations (F.42-43) in [42])

(31) $$\begin{array}{ccc} E_{2}^{1}\left(\rho ,\mu ,\nu \right)=L[\sum_{k=1}^{3}\frac{x_{k}^{2}}{\left(\wedge -a_{k}^{2}\right)}+1]; & E_{2}^{2}\left(\rho ,\mu ,\nu \right)=L^{\prime }[\sum_{k=1}^{3}\frac{x_{k}^{2}}{\left(\wedge^{\prime }-a_{k}^{2}\right)}+1] & \end{array}$$

where the two parameters in Equation (31) are defined below as

$$L=\left(\wedge -a_{1}^{2}\right)\left(\wedge -a_{2}^{2}\right)\left(\wedge -a_{3}^{2}\right),\;L^{\prime }=\left(\wedge^{\prime }-a_{1}^{2}\right)\left(\wedge^{\prime }-a_{2}^{2}\right)\left(\wedge^{\prime }-a_{3}^{2}\right).$$

Note also that $E_{2}^{1}\left(a_{1},\mu ,\nu \right)=$$L\sum_{k=1}^{3}\frac{\wedge x_{k}^{2}}{a_{k}^{2}\left(\wedge -a_{k}^{2}\right)}$ and $E_{2}^{2}L^{\prime }\sum_{k=1}^{3}\frac{\wedge^{\prime }x_{k}^{2}}{a_{k}^{2}\left(\wedge^{\prime }-a_{k}^{2}\right)}$.

Substituting Equation (28) back into Equation (29) and Equation (30) and collecting ${\left(x_{k}/a_{k}\right)}^{2}$ like terms, finally leads to

(32) $$\begin{array}{ccc} A_{k}^{\left(n\right)}=-B^{\left(n\right)}\frac{T_{k}^{\left(n\right)}(a_{1})}{{\left(a_{1}a_{k}\right)}^{2}{\dot{I}}_{1}^{k}(a_{1})}=-\frac{E_{0}^{2}}{6}K^{\left(n\right)}\frac{T_{k}^{\left(n\right)}(a_{1})}{{\left(a_{1}a_{k}\right)}^{2}{\dot{I}}_{1}^{k}(a_{1})}; & & \left(k,n=1,2,3\right) \end{array}$$

where the geometric second-order dimensionless tensor $T_{k}^{\left(n\right)}$ in Equation (32) is defined below as (since $E_{2}^{1}(\rho )=\rho^{2}-a_{1}^{2}+\wedge$ and $E_{2}^{2}(\rho )=\rho^{2}-a_{1}^{2}+\wedge^{\prime }$):

(33) $$T_{k}^{\left(n\right)}(\rho )=a_{1}\{\frac{{\dot{F}}_{0}^{1}(\rho )}{F_{0}^{1}\left(a_{1}\right)}-\frac{1}{\left(\wedge -\wedge^{\prime }\right)}[\frac{{\dot{F}}_{2}^{1}(\rho )L}{F_{2}^{1}\left(a_{1}\right)\left(\wedge -a_{k}^{2}\right)\left(\wedge -a_{n}^{2}\right)}-\frac{{\dot{F}}_{2}^{2}(\rho )L^{\prime }}{F_{2}^{2}\left(a_{1}\right)\left(\wedge^{\prime }-a_{k}^{2}\right)\left(\wedge^{\prime }-a_{n}^{2}\right)}]\}.$$

The above expression can be further simplified by using the identities Equations (G.19-20) in [38], as demonstrated in the next section. Once the ‘streaming’ (DC) part of the non-linear ACEO velocity field around a perfectly polarizable ellipsoidal particle forced by an AC uniform electric field, has been established, one can also determine the vorticity field, by taking the curl of Equation (27) which renders

(34) $${\left(\nabla \times {\vec{u}}^{\left(n\right)}\right)}_{i}=2a_{1}^{2}\sum_{k=1}^{3}\varepsilon_{ikj}A_{k}^{\left(n\right)}x_{k}\frac{\partial I_{1}^{k}\left(\rho \right)}{\partial x_{j}}$$

where $\varepsilon_{ijk}$ denotes the permutation (Levi-Chivita) tensor. This completes the ACEO analysis for general tri-axial ellipsoidal particles.

## 5. Spheres and Spheroids as Limiting ACEO Cases

Using the above methodology, one can readily obtain the corresponding expressions for the case of a perfectly symmetric conducting spherical particle of radius a as a limiting case, by simply letting $a=a_{1}=a_{2}=a_{3}=1$ in the above formulation. For this purpose it is convenient to employ a spherical coordinate system (R,θ,φ) such that $x_{1}=R\mu$, $x_{2}+ix_{3}=R{\left(1-\mu^{2}\right)}^{1/2}e^{i\varphi }$ where μ=cosθ. We also recall that a general ‘exterior’ spherical harmonic (satisfying Laplace’s equation) can be expressed in term of the associate Legendre function $P_{n}^{m}\left(\mu \right)$ as $R^{-\left(n+1\right)}P_{n}^{m}\left(\mu \right)e^{im\varphi }$ for n=0,1,2… and m≤2n+1. In particular, for a spherical particle of unit radius which is exposed to a uniform AC field acting in the $x_{1}$ direction (n=1), we note following Equation (21) that due to symmetry k=n=1 and thus according to Equation (22),

(35) $$\begin{array}{ccc} \psi_{1}\left(R,\mu \right)=\frac{a^{2}P_{1}^{0}\left(\mu \right)}{R^{2}}=\frac{a^{2}x_{1}}{R^{3}}; & \Phi^{\left(1\right)}\left(R,\mu \right)= & \frac{a}{R}+\frac{2a^{3}}{3}\frac{P_{2}^{0}\left(\mu \right)}{R^{3}} \end{array}$$

where $\Phi^{\left(1\right)}$ in Equation (35) satisfies $\Phi^{\left(1\right)}(a,\mu )=\mu^{2}+2/3$ and ${\frac{\partial \Phi^{\left(1\right)}}{\partial R}|}_{R=a}=-3x_{1}^{2}/a^{3}$. Furthermore, by substituting Equation (35) into Equation (27) and using $P_{2}^{0}\left(\mu \right)=\frac{1}{2}\left(3\mu^{2}-1\right)$, one gets

(36) $${\vec{u}}^{\left(1\right)}\left(x_{1},x_{2},x_{3}\right)=a^{2}A_{1}^{\left(1\right)}x_{1}^{2}\nabla \left(\frac{1}{R^{3}}\right)+a^{2}B^{\left(1\right)}\nabla \left[\frac{1}{R}+\frac{a^{2}\left(3x_{1}^{2}-R^{2}\right)}{3R^{5}}\right].$$

The impermeability requirement that ${\vec{u}}^{\left(1\right)}\cdot \vec{n}=0$ on R=a implies then that $B^{\left(1\right)}=-A_{1}^{\left(1\right)}$.

Finally, in order to determine the coefficient $A_{1}^{\left(1\right)}$ by imposing the velocity slippage boundary condition Equation (9), it is necessary first to express (in a similar manner to Equation (15)) the electric potential for a spherical geometry as

(37) $$\begin{array}{ccc} \chi_{1}\left(R,\theta ,\varphi \right)=-2E_{0}\left(R/a-\frac{C_{1}}{R^{3}}\right)\cos\theta ; & & \end{array}$$

Substituting next Equation (37) in Equation (17), namely

(38) $$\begin{array}{ccc} \frac{\partial \chi_{1}}{\partial R}=\frac{i\Omega^{*}}{a}\chi_{1}; & & \Omega^{*}=\frac{\omega a^{2}\lambda_{0}}{D} \end{array}\sqrt{1+\frac{i\omega {\left(a\lambda_{0}\right)}^{2}}{D}}$$

and recalling that for a spherical shape $I_{1}^{1}(a)=1/a^{3}$, one finds from Equations (19) and (20), that

(39) $$\begin{array}{ccc} C_{1}=a^{3}\frac{i\Omega^{*}-1}{i\Omega^{*}+2}; & K^{\left(1\right)}=\frac{9}{4+{\left|\Omega^{*}\right|}^{2}}; & \chi_{1}\left(1,\theta ,\varphi \right)=-\frac{6(x_{1}/a)E_{0}}{2+i\Omega^{*}} \end{array}.$$

The HS velocity slip condition Equation (7) then yields for the tangential velocity component on R=a,

(40) $$u_{\theta }^{\left(1\right)}=\frac{(9/2)E_{0}^{2}}{4+{\left|\Omega^{*}\right|}^{2}}\frac{\partial }{\partial \theta }{\left(\frac{x_{1}}{a}\right)}^{2}$$

Comparing Equation (40) against Equation (36) promptly renders $B^{\left(1\right)}=\frac{(9/2)E_{0}^{2}}{4+{\left|\Omega^{*}\right|}^{2}}$.

The sought expression for the non-dimensional polarization induced velocity past a spherical metallic particle can then be found from Equation (36) by replacing $\frac{P_{2}^{0}\left(\mu \right)}{R^{3}}=\frac{1}{2}\frac{\partial^{2}}{\partial x^{2}}\left(\frac{1}{R}\right)$ as

(41) $${\vec{u}}^{\left(1\right)}\left(x_{1},x_{2},x_{3}\right)=\frac{(9/2)E_{0}^{2}a^{2}}{4+{\left|\Omega^{*}\right|}^{2}}\left\{x_{1}^{2}\nabla \left(\frac{1}{R^{3}}\right)-\left(1+\frac{a^{2}}{3}\frac{\partial^{2}}{\partial x_{1}^{2}}\right)\nabla \left(\frac{1}{R}\right)\right\}$$

where the last term represents the Faxen [45] correction. The quadrupole-type ACEO velocity field Equation (41) preserves axial-symmetry about the $x_{1}$ axis and thus may be also obtained directly from the following Stokes stream-function:

(42) $$\Psi \left(R,\theta \right)=\frac{(9/2)E_{0}^{2}}{4+{\left|\Omega^{*}\right|}^{2}}\left(\frac{a^{2}}{R^{2}}-1\right)\sin^{2}\theta \cos\theta$$

Implying that the corresponding radial and tangential velocity components are given by

(43) $$\begin{array}{l} \begin{array}{cc} V_{R}\left(R,\theta \right)=\frac{(9/2)E_{0}^{2}}{4+{\left|\Omega^{*}\right|}^{2}}\left(\frac{a^{4}}{R^{4}}-\frac{a^{2}}{R^{2}}\right)\left(3\cos^{2}\theta -1\right) & \end{array}\\ V_{\theta }\left(R,\theta \right)=\frac{9E_{0}^{2}}{4+{\left|\Omega^{*}\right|}^{2}}\frac{\sin\theta \cos\theta }{{\left(R/a\right)}^{4}}. \end{array}$$

The ACEO velocity field in Equation (43) decays to zero for large values of $\Omega^{*}$ and has a maximum at $\Omega^{*}=0$. The analytic expression for the Stokes stream-function Equation (42) and the associated velocity components Equation (43) for an induced ACEO flow past a sphere are identical with those given in [32]. A Cartesian leading-order representation of the same quadrupole-type velocity field in the DC limit (i.e., $\Omega^{*}=0$) which is similar to Equation (41) (but without the last Faxen’s term), has been also obtained in [46]. The ACEO flow along the axis of symmetry $\left(x_{1}\right)$ is directed towards the conducting particle with stagnation points at $x_{1}=\pm a$ and a maximum velocity on the major axis at $x_{1}=\pm \sqrt{2}a.$ In a similar manner, the velocity field around the other two semi- axes, is directed away from the particle, exhibiting again a maximum at $x_{2},x_{3}=\pm \sqrt{2}a$ with a total of six stagnation points located on the conducting particle.

Before concluding this section, let us apply a similar methodology to analytically determine the corresponding ACEO velocity field for the case of a prolate spheroid. For this purpose we employ a spheroidal coordinate system (ζ,μ,φ), such that $x_{1}=c\mu \zeta$, $x_{2}+ix_{3}=c{\left(1-\mu^{2}\right)}^{1/2}{\left(\zeta^{2}-1\right)}^{1/2}e^{i\varphi }$ and 2c denotes the distance between the two spheroidal foci. For the axisymmetric case (m=0) where the ambient electric field is directed along the $x_{1}$ axis, the harmonic functions in Equation (21) can be defined in terms of the corresponding Legendre functions of the first $P_{n}(\mu )$ and second kind $Q_{n}\left(\zeta \right)$, as $\psi_{1}=(x_{1}/c)I_{1}(\zeta )$ with $I_{1}(\zeta )=Q_{1}(\zeta )/\zeta$ and $\Phi^{\left(1\right)}=Q_{0}(\zeta )+\beta P_{2}(\mu )Q_{2}(\zeta )$. Here $\beta =2{\dot{Q}}_{1}(\zeta_{0})/{\dot{Q}}_{2}(\zeta_{0})$ is a coefficient determined from imposing the impermeability conditions on $\zeta =\zeta_{0}$. For the purpose of illustration, we also provide below an analytic expression for the ACEO velocity component which prevails along the axis of symmetry $x_{1}=c\zeta$ of a prolate spheroid, namely

(44) $$u^{\left(1\right)}(x_{1},0,0)=\frac{E_{0}^{2}K^{\left(1\right)}\zeta_{0}{\dot{Q}}_{2}(\zeta_{0})}{3Q_{2}(\zeta_{0})}\left[\frac{{\dot{Q}}_{2}\left(x_{1}/c\right)}{{\dot{Q}}_{2}(\zeta_{0})}+\frac{{\dot{Q}}_{0}(x_{1}/c)}{2{\dot{Q}}_{0}(\zeta_{0})}-\frac{3}{2}{\left(x_{1}/c\zeta_{0}\right)}^{2}\frac{{\dot{I}}_{1}(x_{1}/c)}{{\dot{I}}_{1}(\zeta_{0})}\right].$$

**Figure 2:**
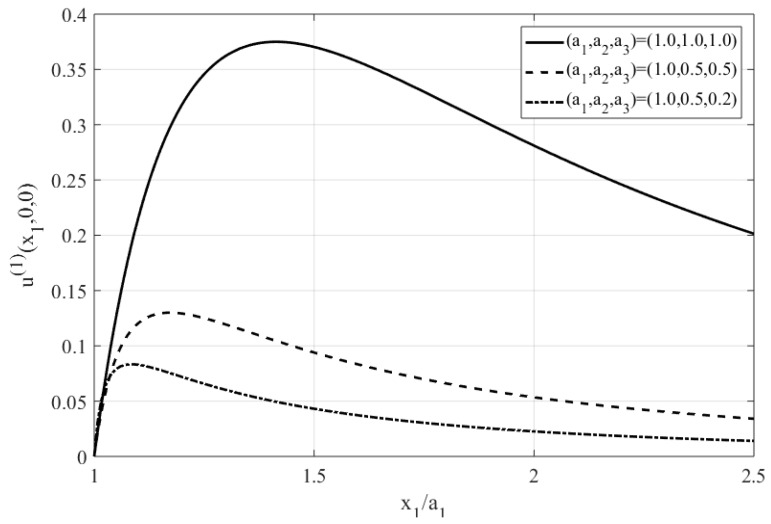
A plot of the normalized velocity component (the term in the square bracket of Equation (48)) versus $x_{1}/a_{1}$ for various morphologies (i.e., sphere, spheroid and tri-axial ellipsoid).

The frequency dependent parameter $K^{\left(1\right)}(\Omega^{*},\zeta_{0})$ in Equation (44) for a prolate spheroid, can be obtained directly from Equations (18) and (19) as $1/K^{\left(1\right)}=(\zeta_{0}^{2}-1)\{{\left[\Omega^{*}Q_{1}(\zeta_{0})\right]}^{2}+{\left[{\dot{Q}}_{1}(\zeta_{0})\right]}^{2}\}$. It can be also easily verified that the longitudinal velocity (i.e., $u_{1}^{\left(1\right)}=0$) indeed vanishes at $x_{1}=\pm c\zeta_{0}$ (stagnation points). In the limit of a sphere of radius a one gets [40] $\zeta_{0}\to \infty$, c→0 such that $a=c\zeta_{0}$. Under these limits, Equation (44) renders $u_{1}^{\left(1\right)}(x_{1},0,0)=E_{0}^{2}K^{\left(1\right)}\left[{\left(a/x_{1}^{}\right)}^{4}-{\left(a/x_{1}\right)}^{2}\right]$ which agrees with Equation (43) with $K^{\left(j\right)}$ given by Equation (39). Equation (44) is found useful in the next section when addressing the problem of controlled positioning of free molecules (QD) or biosensors by means of AC electrokinetics. Figure 2 shows the variation of the axial velocity component $u_{1}^{\left(1\right)}(x_{1},0,0)$ along the major axis for various shapes (i.e., sphere, prolate spheroid and tri-axial ellipsoid), displaying a boundary-layer type behavior in the vicinity of the nearby stagnation points.

## 6. Positioning and Manipulation of Quantum Dots

The technical problem addressed in this work is the manipulation and positioning of a single QD at some prescribed small distance from a non-spherical micron-size object and the ability to simply control this distance by adjusting and tuning the forcing frequency of the ambient field. The polarizable entity is regarded in this context as a nano-antenna (NA). We consider here a single (spherical) molecule (QD) of radius *b* which is located at a distance $\tilde{d}$ from a stagnation point next to a polarizable tri-axial ellipsoid (Figure 1). In order to obtain maximum plasmon electromagnetic enhancement [14,15,16,17,18,19], the QD must be placed next to the NA such that $\tilde{d}\leq a_{1}{}_{,2}$ is of the same order of magnitude as the size of the fluorophore (i.e., few tenths of a nm).

As demonstrated (see for example [28,29,30]) active manipulation and positioning (trapping) of a sole QD or single molecule, can be effectively achieved by means of AC electrokinetics. The freely suspended QD is generally exposed to both dielectrophoresis (DEP) force due to the presence of a nearby NA as well as to electroosmotic (ACEO) flow. The short-range DEP force, resulting from the field non-uniformity around the NA at the location of the spherical QD, is given by [21]

(45) F→DEP=2πb3εRe{K(ω)}∇|Erms|2

where ε (real) is the typical medium (solute) permittivity, *E_rms_* denotes the root-mean square of the electric field and Re{K(ω)} represents the real part of the frequency-dependent CM coefficient of the fluorophore. Following Jones [21], the CM coefficient can be expressed in terms of the complex dielectric constants of the molecule (m) and fluid (f) as ${\underline{\varepsilon }}_{f,m}=\varepsilon_{f,m}+\sigma_{f,m}/i\omega$, where $\varepsilon_{f,m}$ and $\sigma_{f,m}$ represent the permittivity and conductivity coefficients (real) of the corresponding phase. For example, for a spherical QD one gets $K(\omega )=({\underline{\varepsilon }}_{p}-{\underline{\varepsilon }}_{f})/(2{\underline{\varepsilon }}_{f}+{\underline{\varepsilon }}_{p})$, implying that the CM coefficient can change sign depending on the relative polarizability between fluorophore (m) and electrolyte (f). In general, at relatively low frequencies it is plausible to assume that Re{K(ω)}∼1 for a ‘metallic’ (perfect conductor) QD and Re{K(ω)}∼0.5 for ‘organic’ (biological) or synthetic molecules. Thus, the dielectrophoretic force can be either positive, i.e., directed towards the NA (pDEP) or be repelled from it (nDEP) when acting in the opposite direction (away from the NA). Free QDs can be generally regarded as simple tracers in the sense that they follow the induced ACEO velocity field ${\vec{v}}_{ACEO}$ around the polarizable NA. Hence, the Stokes drag experienced by a spherical QD, is simply given by ${\vec{F}}_{ACEO}=6\pi \eta b{\vec{v}}_{ACEO}$, where η denotes the dynamic viscosity of the solute.

**Figure 3:**
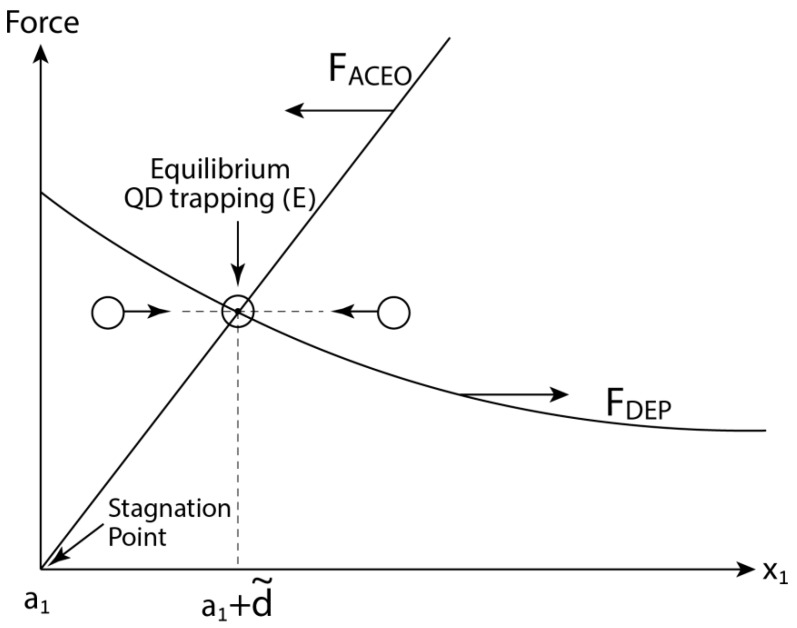
The competing effects between DEP and ACEO forces exerted on a free QD located on the longitudinal axis and demonstrating the existence of an equilibrium (saddle-like) trapping point (E) at a distance $\tilde{d}$ from the nano-antenna.

Let us consider for example the particular configuration depicted in Figure 1. The AC ambient electric field is collinear with the longitudinal $x_{1}$ axis and thus the induced quadrupole ACEO velocity field is directed towards the two poles (stagnation points) of the NA located on the major axis (suction) and away from the NA (ejection), along the other two semi-axes [23,32,33,34,35,36]. Thus, depending on QD location, ${\vec{F}}_{ACEO}$ can be either positive (directed away from the NA) for QDs lying on the semi-axes, or negative (directed towards the NA), for QDs placed along the major axis. Under certain conditions and depending on the sign of CM, ${\vec{F}}_{ACEO}$ and ${\vec{F}}_{DEP}$ can augment or counteract each other. For this reason, a single QD can be trapped and positioned at a certain point at a small distance $\tilde{d}$ from the NA, providing the DEP and ACEO forces are equal in magnitude and act in opposite directions $\left({\vec{F}}_{ACEO}+{\vec{F}}_{DEP}=0\right)$, as illustrated for example in Figure 3. Such a trapping point can be either stable to perturbations along all three orthogonal directions here defined as a stable trapping (S), or only for agitation along one or two directions, which is labeled here as equilibrium (saddle-like) trapping (E). In addition to the above two trapping cases, there is yet a third possible physical stable labeled as wall trapping (W), which generally occurs around one of the stagnation points of the NA. These three trapping scenarios will be further analyzed and demonstrated below for the case of ellipsoidal NA and spherical QD.

For the purpose of illustration, we first examine the possibility (Figure 1) of trapping a free QD at a certain point $x_{1}=a_{1}+\tilde{d}$ placed along the major $x_{1}$ axis of a tri-axial ellipsoid when the AC ambient field is acting in the same direction (n=1). The induced ACEO flow is then directed towards the NA (i.e., $u_{1}^{\left(1\right)}(x_{1},0,0)<0$). Recalling that ${\vec{u}}^{\left(1\right)}$ Equation (27) is made dimensionless with respect to $\varepsilon \varphi_{T}^{2}/a_{1}\eta =\varepsilon E_{0}^{2}a_{1}/\eta$, a simple force balance between DEP and ACEO then yields

(46) 13b2a1Re{K(ω)}∇|Erms|2=−u→(1)(x1,0,0),

since along the $x_{1}$ axis, $\mu =h_{2},\;\upsilon =h_{3}$ and thus following Equation (10) one gets $x_{1}=\rho \geq a_{1}$. It is important to note that trapping on the $x_{1}$ axis is possible only for the case of nDEP, namely when, Re{K(ω)}<0 (organic or artificial QDs), where both sides of Equation (46) are positive. The ACEO velocity component along the $x_{1}$ axis can then be obtained directly from Equations (20), (27), (28), and (32) as

(47) $$u_{1}^{\left(1\right)}(x_{1},0,0)=a_{1}^{2}A_{1}^{\left(1\right)}x_{1}^{2}{\dot{I}}_{1}^{1}(x_{1})+a_{1}B^{\left(1\right)}\frac{\partial \Phi^{\left(1\right)}}{\partial x_{1}}=\frac{K^{\left(1\right)}E_{0}^{2}}{6}\left[a_{1}\frac{\partial \Phi^{\left(1\right)}}{\partial x_{1}}-{\left(\frac{x_{1}}{a_{1}}\right)}^{2}\frac{{\dot{I}}_{}^{1}{}_{1}\left(x_{1}\right)}{{\dot{I}}_{}^{1}{}_{1}\left(a_{1}\right)}T_{1}^{\left(1\right)}\left(a_{1}\right)\right].$$

Equation (47) can be further simplified by making use of Equations (14) and (30), finally leading to

(48) $$u_{1}^{\left(1\right)}(x_{1},0,0)=\frac{K^{\left(1\right)}E_{0}^{2}T_{1}^{1}\left(a_{1}\right)}{6}\left[\frac{T_{1}^{\left(1\right)}\left(x_{1}\right)}{T_{1}^{\left(1\right)}\left(a_{1}\right)}-\frac{a_{2}a_{3}}{\sqrt{\left(x_{1}^{2}-h_{2}^{2})(x_{1}^{2}-h_{3}^{2}\right)}}\right]$$

where $T_{1}^{\left(1\right)}$ in Equation (48) is defined in Equation (33). Note also that both points $\left(\pm a_{1},0,0\right)$ are indeed stagnation points since $u_{1}^{\left(1\right)}(\pm a_{1},0,0)=0$ and $u_{2,3}^{\left(1\right)}(x_{1},0,0)=0$ due to symmetry.

What remains in order to determine the DEP force exerted on the QD is to compute the gradient of $E_{rms}^{2}$ given in Equation (46) and evaluate it at the potential trapping point $\left(x_{1},0,\;0\right)$. Towards this goal we recall following Equations (1) and (15) that under the assumption of a thin EDL, ${\vec{E}}_{rms}=-\nabla \varphi \approx \frac{1}{2}\nabla \chi_{1}$ and thus

(49) |Erms/E0|2≈1−2Re{C1}∂∂x1[x1I11(x1)]+|C1∂∂x1[x1I11(x1)]|2>0; for x1>a1

Substituting next $E_{1}^{1}\left(t\right)=t$ in Equation (14), one can evaluate $I_{1}^{1}(t)$ and show that

(50) $$\nabla {\left|E_{rms}/E_{0}\right|}^{2}=-\frac{6}{\sqrt{\left(x_{1}^{2}-h_{2}^{2})(x_{1}^{2}-h_{3}^{2}\right)}}\left(\frac{1}{x_{1}^{2}-h_{2}^{2}}+\frac{1}{x_{1}^{2}-h_{3}^{2}}\right)Ρ(\Omega^{*}).$$

The parameter $Ρ(\Omega^{*})$ evaluated near the stagnation points $\left(\pm a_{1},0,0\right)$ is defined as

(51) Ρ(Ω∗)=2[Re{C1}−|C1|2ddx1[x1I11(x1)]x1=a1]=∀K(1)2π|Ω∗|2

where $C_{1}$ and $K^{\left(1\right)}$ are defined respectively in Equations (19) and (20). The final expression for the trapping point (*x*_1_,0,0) i is then obtained by substituting Equations (47), (49), and (50) into (45) resulting in

(52) T1(1)(x1)(x12−h22)(x12−h32)a2a3−T1(1)(a1)=4b2|Ω∗|2Re{K(ω)}(1x12−h22+1x12−h32).

Equation (52) is the sought relation between the trapping distance $x_{1}=a_{1}+\tilde{d}$ of a single free fluorophore placed near a tri-axial ellipsoid and the detuning frequency parameter $\Omega^{*}$ where nDEP and ACEO effects precisely cancel each other. The value of $\tilde{d}$ depends on the ellipsoid geometry and the radius *b* of the QD, as well as on the forcing frequency and chemical properties of the ambient electrolyte (i.e., its molar concentration and effective diffusivity). The precise location (position) of a free QD next to a polarizable ellipsoid can be thus adjusted by tuning the AC frequency which serves here as the control parameter. Since (see [14,15,16]) the ‘spacing’ $\tilde{d}$ is generally small compared to $a_{1}$, the left hand side of Equation (52) can be approximated to leading-order using Equations (14), (33) and the relations given in ( see Equations (G.18-19) in [42]) by $-(\tilde{d}/a_{1})\Delta (a_{1})$, where

(53) $$\begin{array}{l} \Delta (a_{1})=-\frac{a_{1}}{a_{2}a_{3}}\frac{d}{dx_{1}}{\left[T_{1}^{\left(1\right)}(x_{1})\sqrt{\left(x_{1}^{2}-h_{2}^{2})(x_{1}^{2}-h_{3}^{2}\right)}\right]}_{x_{1}=a_{1}}=3{\left(\frac{a_{1}}{a_{2}a_{3}}\right)}^{2}[2a_{1}^{2}+a_{2}^{3}+a_{3}^{2}] \end{array}$$

so that the trapping distance $\tilde{d}$ (a saddle-like equilibrium point) can be finally expressed using Equation (52) as

(54) d˜/a1∼−43(ba1)2(a22+a322a12+a22+a32)|Ω∗|2Re{K(ω)}.

In the limit of a perfectly symmetric sphere of radius *a*, $h_{2}=h_{3}=0\;$ and $I_{1}^{1}(a)=1/a^{3}.$ Thus, the right-hand-side of Equation (52) simply reduces to 8(b/a)2|Ω∗|2Re{K(ω)}(a2/x12)<0. Following Equation (33), it can then be shown that $T_{1}^{\left(1\right)}(x_{1})={\dot{F}}_{0}^{\left(1\right)}(x_{1})/F_{0}^{\left(1\right)}(a_{1})-2{\dot{F}}_{2}^{1}(x_{1})/F_{2}^{1}(a_{1})$, since in this case $F_{2}^{1}(x_{1})=F_{2}^{2}(x_{1})$. Furthermore, we note following Equation (14) that $F_{0}^{1}(x_{1})=1/x_{1}$ and $F_{2}^{1}(x_{1})=1/x_{1}^{3}$. Hence, one finds that $T_{1}^{\left(1\right)}(x_{1})=-{\left(a/x_{1}\right)}^{2}+6{\left(a/x_{1}\right)}^{4}$ and for a spherical NA the left-hand side of Equation (52) simply renders $-6[1-{\left(a/x_{1}\right)}^{2}]$. Substituting these values back in Equation (52), we finally get the following exact relation between the trapping distance $\tilde{d}$ and the frequency control parameter $\Omega^{*}$ near a spherical NA:

(55) (ax1)2=(1+d˜/a)−2=1+43(ba)2|Ω∗|2Re{K(ω)}

implying that d˜/a∼−23(ba)2|Ω∗|2Re{K(ω)} for $\tilde{d}/a\ll 1$. On the other hand, when using the above expression for $T_{1}^{\left(1\right)}(x_{1})$ in Equation (53), it yields $\Delta (a)=-\frac{1}{a}\frac{d}{dx_{1}}{\left[x_{1}^{2}T_{1}^{\left(1\right)}(x_{1})\right]}_{x_{1}=a}=12$, which by virtue of Equation (52), yields the exact relation (55) for a spherical morphology.

**Figure 4:**
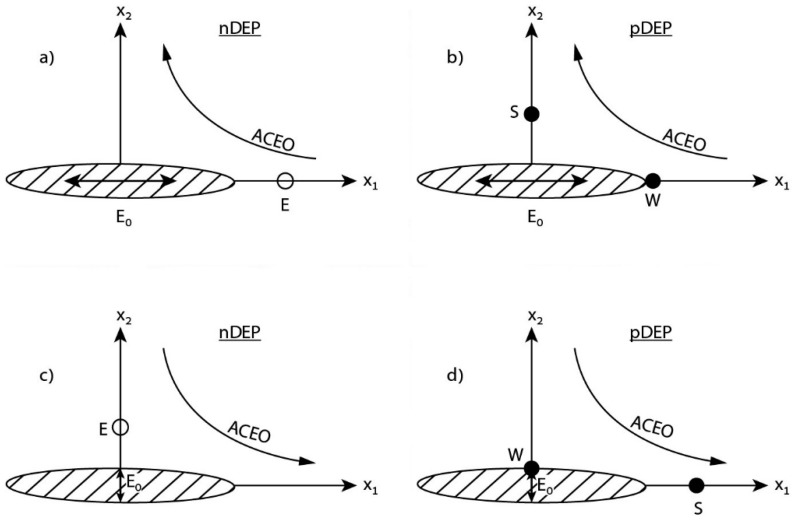
Four scenarios describing stable (S) and equilibrium (E) saddle-like trapping points in the fluid as well as stable wall (W) stagnation trapping for different orientations of the ambient AC electric field and sign of the Clausius Mossotti (CM) coefficient of a free QD.

Regarding stability issues of these particular (nDEP) trapping points at $\left(x_{1},0,0\right)$ where $x_{1}=\pm (a_{1}+\tilde{d})$, one finds that they are indeed stable for perturbations in the longitudinal $x_{1}$ direction, however closer scrutiny indicates that they are unstable for perturbations along the transverse $x_{2,3}$ semi-axes and thus by definition they are labeled here as equilibrium (saddle-like) points (E), as depicted in Figure 4a. Nevertheless, for the same longitudinal electric forcing but under pDEP conditions (Figure 4b), there exist four stable (S) trapping points located on the semi-axes $x_{2,3}$ and two stable wall (W) trapping points at the tip stagnation points $\left(\pm a_{1},0,0\right)$. For the case of a transverse electric forcing (acting along the $x_{2,3}$ direction), repeating the same analysis reveals that for nDEP, there are four equilibrium (E) points lying on the semi-axes (Figure 4c). Finally, for the same transverse forcing but under pDEP predicaments, one finds two stable (S) trapping points located along the major $x_{1}$ axis and four stable wall (W) trapping points coinciding with the corresponding $x_{2,3}$ stagnation points (Figure 4d). Thus, in principle, one can control the position $\tilde{d}$ of a free QD lying next to an ellipsoidal NA, by simply adjusting the forcing frequency ω of the ambient field. The particular form of the corresponding transfer function (i.e., distance vs. frequency) depends on the diffusivity and concentration of the electrolyte (through the Debye scale) and on the sign of the CM coefficient (namely, nDEP or pDEP).

To conclude this section and for the purpose of illustration, we provide a simple demonstration of the equilibrium (E) nDEP trapping scenario depicted in Figure 4a and given explicitly by Equation (54) for determining the relation between the frequency ω to be applied for positioning a free QD of radius b at a prescribed distance $\tilde{d}$ from a polarizable ellipsoidal NA. In particular, let us choose (see for example [14,15,16,17,18,19]) the following parameters; $a_{1}=1\mu m,\;\;\;a_{2}=0.5\mu m,\;\;\;a_{3}=0.2\mu m{,}_{}^{}$ b=5nm and $\tilde{d}=13\mathrm{nm}$ (i.e., minimum NA/QD clearance of $\tilde{d}-b∼8\mathrm{nm}$). As a reference frequency, we use the Maxwell–Wagner value [23,24] $\omega_{MW}=D/\lambda_{0}^{2}$, defined in terms of the solute diffusivity D and the EDL thickness (Debye scale) $\lambda_{0}=\sqrt{D\varepsilon_{f}/\sigma_{f}}$ and use archetypal value of [34] $\lambda_{0}∼25\mathrm{nm}$ for DI water. A typical value of the real part of the CM coefficient [47] (prevailing at forcing frequencies above 500 kHz), is close to 0.9 for metallic colloids (pDEP) and about −0.46 for dielectric (silica) or synthetic (nDEP) particles immersed in DI water. Hence, in accordance with Figure 4a, let us assume a non-conducting organic (biological) dye molecule lying in a solute together with Re{K(ω)}∼−0.5. Following Equation (17) and (54), one gets $\omega /\omega_{MW}{\left[1+{\left(\omega /\omega_{MW}\right)}^{2}\right]}^{1/4}∼\frac{1}{r}(\frac{\lambda_{0}}{b})\sqrt{\frac{3\tilde{d}(2a_{1}^{2}+a_{2}^{2}+a_{3}^{2})}{2a_{1}(a_{2}^{2}+a_{3}^{2})}}$, where the ‘slenderness’ parameter *r* defined in Equation (18) can be selected as $r∼a_{1}/a_{3}>1$. Finally, using the above parameters for $D=10^{-9}m^{2}/\sec$ (DI water), one gets $\omega /\omega_{MW}∼0.4$ or a trapping frequency equals to f=ω/2π∼100kHz.

## 7. Summary and Discussions

Single molecule (quantum dot) fluorescence imaging and spectroscopy are very powerful present-day techniques often used in various branches of physics, chemistry, biology and material sciences that are based on controlling and positioning of free fluorophores next to nano-antennas (NA) of non-spherical shapes. It has been demonstrated that changing the morphology of the NA from say spherical to a tri-axial ellipsoid, can lead to substantial enhancement of the spontaneous emission radiative decay rates of a QD depending on its specific distance from the tip of the NA. Furthermore, there exists an optimal QD spacing for a maximum enhancement. Motivated by this physical finding, we chose to analytically study the possibility of accurately controlling the position of a free QD placed next to an ellipsoidal NA using common AC electrokinetic procedures by means of adjusting the frequency of the applied electric field. Stable or equilibrium (saddle-like) QD trapping points can then be obtained, both in the fluid or at the NA stagnation points, depending on the direction of the ambient AC field and sign of CM. Thus, the hydrodynamic effects associated with induced-charge electro-osmosis (ACEO) and those resulting from dielectrophoresis (DEP), can either cancel or augment each other.

In the first part of the paper, we analyze the nonlinear ACEO flow problem about a polarizable tri-axial ellipsoidal entity. Employing the common ‘weak field’ and small Dukhin number assumptions, we use the traditional Poisson–Nernst–Planck (PNP) formulation and the ‘thin’ EDL (small Debye scale) conjecture to determine the polarization-induced electric field around the NA. Closed form expressions are then obtained for the non-uniform electric field as well for the charge density distribution in the solute in terms of ellipsoidal harmonics and Lamé functions. Substituting the polarization-induced Coulombic force term in the non-homogeneous Stokes momentum equation, renders explicit expressions for the induced ACEO velocity, pressure, and vorticity fields in the symmetric (1:1) electrolyte surrounding the ellipsoid. It is shown that the new solution thus found for tri-axial ellipsoidal morphologies, reduces to the well-known solution for a perfectly symmetric spherical particles [33,36,46].

The quadrupole- type ACEO flow field in the solute is found by enforcing the HS velocity slip condition on the ellipsoid (Figure 1). The velocity component along the major $x_{1}$ axis decays to zero far from the NA and vanishes at the stagnation points $x_{1}=\pm a_{1}$, exhibiting a local maximum close to the ellipsoidal object (Figure 2.) depending on its morphology. For a spherical shape, the ACEO velocity has a local peak at $x_{1}/a=\pm \sqrt{2}$ and this value decreases as the ellipsoid gets flatter (i.e., smaller values of $a_{3}/a_{1}$) as depicted in Figure 2. Near the stagnation points the velocity displays a boundary-layer type behavior and increases (linearly) with the distance $\tilde{d}=x_{1}-a_{1}$.

Once the ACEO hydrodynamic problem about a perfectly conducting (metallic) tri-axial ellipsoid has been solved (Section 3), we explore in the second part of the text (Section 4) the possibility of using it as a nano-antenna for trapping single molecules (QD) placed near the stagnation points (Figure 3) by means of conventional AC electrokinetics. The idea here is to treat the QD, or a single spherical fluorophore (radius of few nm), as a free tracer which is carried by the ambient ACEO flow field and thus experiences a common Stokes drag. Thus, for a free QD located on the $x_{1}$ axis (collinear with the applied AC field as shown in Figure 1), the ACEO (Stokes) force is directed towards the stagnation points (Figure 3). Resisting (or adding) to this long-range hydrodynamic force, there is a short-range DEP force acting on the QD (resulting from the non-uniformity of the electric field around the NA). The DEP force can act along the $x_{1}$ axis (nDEP) or in the opposite direction (pDEP), depending of the sign of the real part of the CM coefficient of the QD. For most organic (biological) molecules or synthetic (polymeric) QDs embedded in a conducting electrolyte within the Maxwell–Wagner frequency range, Re{K(ω)}<0 and thus a negative (nDEP) force prevails. In such a case, the DEP force is acting in the opposite direction to the ACEO force (Figure 3) and trapping of a free QD can be attained at a particular distance $\tilde{d}$ from the tip (stagnation point) of the NA where these two opposing forces balance each other. This particular trapping point is apparently ‘stable’ only for perturbations in the longitudinal $x_{1}$ direction (see also Figure 4a) but it appears to be unstable in the transverse $x_{2,3}$ directions. For these reasons and following our notations, we label it as an equilibrium (saddle-like) point (E). Nevertheless, it is interesting to note that by applying the electric field along the semi-axes $x_{2,3}$ and depending on the sign of CM (namely nDEP vs. pDEP), one finds *stable* trapping points in the fluid (S) or at the stagnation points (W) on the semi-axes (see Figure 4.).

The above analysis provides us with an explicit relation between the desired (optimal) QD position $\tilde{d}$ next to an ellipsoidal NA and the forcing frequency *ω* (control parameter). It is found that depending on electrolyte physical properties and QD size, one can use the following approximation $\tilde{d}∼\chi^{2}{\left(1+\chi^{2}\right)}^{1/2}$ for the optimal QD spacing $\tilde{d}(\omega )$ where $\chi =\omega /\omega_{MW}$. Optimal trapping frequencies are usually below the Maxwell–Wagner limit and are thus of the order of few hundred kHz. To conclude, we have theoretically demonstrated the feasibility of using conventional AC electrokinetic techniques as an alternative effective procedure for manipulating, controlling, trapping, and positioning quantum dots or free fluorophores placed next to non-spherical (ellipsoidal) nano-antennas.
